# Patient-reported outcomes in coronary artery disease: the relationship between the standard, disease-specific set by the International Consortium for Health Outcomes Measurement (ICHOM) and the generic health-related quality of life instrument 15D

**DOI:** 10.1186/s12955-021-01841-6

**Published:** 2021-08-28

**Authors:** Laura Lappalainen, Harriet Stenvall, Piia Lavikainen, Heikki Miettinen, Janne Martikainen, Harri Sintonen, Anna-Maija Tolppanen, Risto P. Roine, Juha Hartikainen

**Affiliations:** 1 Kuopio University Hospital, Heart Center C9, P.O. Box 100, 70029 Kuopio, Finland; 2 University of Helsinki, Helsinki, Finland; 3 University of Eastern Finland, Kuopio, Finland

**Keywords:** Coronary artery disease, Health-related quality of life, Health status assessment, Patient-reported outcomes

## Abstract

**Background:**

Patient-reported outcome (PRO) instruments measure health gains, including changes in health-related quality of life (HRQoL). Previous studies have assessed the reliability and relationship of multiple HRQoL instruments in search of the optimal instrument for feasible measurement of PROs. Although the 15D instrument was shown to have the best sensitivity and construct validity among cardiac patients, it is unknown how well it captures relevant disease-specific information scores compared to instruments included in the International Consortium for Health Outcomes Measurement (ICHOM) standard set. The aim of this study was to investigate whether the disease-specific PRO instruments and a generic HRQoL instrument capture disease related symptoms in coronary artery disease (CAD) patients.

**Methods:**

Health status and HRQoL were assessed with the instruments included in the ICHOM standard set: Seattle Angina Questionnaire short-form (SAQ-7), Rose Dyspnea Scale (RDS), two-item Patient Health Questionnaire (PHQ-2), and with the 15D HRQoL instrument at baseline and 1 year from the treatment in a university hospital setting. Spearman correlation and explanatory factor analysis were used to assess the relationship of baseline scores and 1-year change in scores of 297 patients.

**Results:**

At baseline, the overall 15D score and SAQ-physical limitation (SAQ-PL), 15D “breathing” and SAQ-PL, as well as “breathing” and RDS showed moderately strong correlations. The factor interpreted to reflect “Breathing-related physical activity”, based on high loadings of “breathing”, RDS, SAQ-PL, “mobility”, “vitality”, and “usual activities”, explained 19.2% of the total variance. Correlations between 1-year changes in scores were fair. The factor of “Breathing-related physical activity”, with significant loading of RDS, SAQ-PL, “breathing, “usual activities”, “vitality”, “sexual activity”, “mobility”, and disease-specific quality of life explained 20.5% of the total variance in 1-year change in scores. The correlation of angina frequency measured by SAQ-7 and the 15D instrument was poor.

**Conclusions:**

The 15D detects dyspnea and depression similarly to RDS and PHQ-2 but not angina similarly to the SAQ-7. This may call for supplementing the 15D instrument with a disease-specific instrument when studying CAD patients.

**Supplementary Information:**

The online version contains supplementary material available at 10.1186/s12955-021-01841-6.

## Background

Validated patient-reported outcome (PRO) instruments measure health gains, including changes in health-related quality of life (HRQoL), perceived by patients after treatment [1]. Responding to health status assessment questionnaires should be as simple as possible for the respondents. Thus, long, or numerous questionnaires tend to impair response rates [1]. From a healthcare provider´s point of view, a PRO instrument should capture the health status change diversely with minimal resources needed for collection and analysis of the PROs.

In search of the optimal instruments for feasible measurement of PROs in routine care of cardiac patients, previous studies combined multiple HRQoL instruments and assessed their reliability and relationship [2–6]. Several studies have included preference-based generic instruments such as the 15D [7], Assessment quality of life instrument (AQoL), five-dimensional EuroQol instrument (EQ-5D), Health utilities index mark three instrument (HUI3), and short-form six dimension instrument (SF-6D) [4, 8–11]. Of these generic HRQoL instruments, the 15D has demonstrated to have the best sensitivity and construct validity among cardiac patients [4, 10, 11]. However, as 15D is a generic HRQoL instrument, it is unknown whether it can capture the variation in the disease-specific measures in patients with coronary artery disease (CAD).

The International Consortium for Health Outcomes Measurement (ICHOM) CAD working group recommends measurement with a standard set for CAD [12]. The set includes the following instruments: Seattle Angina Questionnaire short-form (SAQ-7) [13] for assessing functional status, angina and disease-specific HRQoL, Rose Dyspnea Scale (RDS) [14] for assessing dyspnea, and the two-item Patient Health Questionnaire (PHQ-2) [15, 16] for evaluating depressive symptoms. The measurement is recommended to be performed at baseline, and one month and 1 year from the treatment. However, only a few studies utilizing the ICHOM standard set of instruments have so far been published [17, 18]. Furthermore, although the standard set includes assessment of disease-specific HRQoL with the SAQ-7, it does not include measurement of generic HRQoL and thus excludes the possibility to compare HRQoL outcomes obtained in CAD to those obtained in other diseases or the general population, and the calculation of quality-adjusted life-years (QALYs).

We investigated how well the generic HRQoL, 15D, captures disease-specific quality of life and symptoms measured by the instruments included in the ICHOM standard set in a routine setting. The analyses were performed using baseline scores and the changes in scores during 1-year follow-up.

## Methods

### The patient recruitment

A total of 397 (Additional file 1: Table S1) CAD patients scheduled for elective index angiography or elective coronary artery bypass grafting (CABG) at the Heart Centre of the Kuopio University Hospital (KUH) between July 2017 and May 2018 self-assessed their health status and HRQoL with the ICHOM standard set of instruments (SAQ-7, RDS, PHQ-2), and the 15D HRQoL instrument before the treatment and at 1 year after treatment. All questionnaires were administered in paper form and the CAD diagnosis was based on a previous CAD diagnosis and findings in the baseline coronary angiography. This analysis was restricted to those 279 (70.3%) patients for whom 1-year change with all four instruments could be calculated. Based on intention to treat, 100 (35.8%) patients received optimal medical therapy (OMT), 155 (55.6%) underwent percutaneous coronary intervention (PCI), and 24 (8.6%) CABG. A total of 118 patients were excluded from the analysis, 69 patients (n = 39 at baseline, n = 30 at 1-year) due to ≥ 1 missing instrument scores, and 49 patients due to non-response at 1-year follow-up (8 of them had died).

### Assessment of health status and health-related quality of life

The seven-item SAQ-7 measures physical limitation (SAQ-PL), angina frequency (SAQ-AF), and disease-specific HRQoL (SAQ-QL) and has a four-week recall period. The SAQ-7 generates a summary score (scale 0–100, 100 = full health, 0 = worst health). The SAQ-AF corresponds to two questions and categorizes angina frequency as following: daily angina (score = 0‒30), weekly angina (score = 31–60), monthly angina (score = 61‒99), and no angina (score = 100). The SAQ-PL corresponds to three questions and SAQ-QL corresponds to two questions. If all three domain scores are missing, the SAQ-7 summary score is not calculated. According to prior work, a change of 5‒8 points in the summary score is considered clinically important [13].

The four-item symptom-specific RDS measures dyspnea level during activity (scale 0‒4, 0 = no dyspnea, 4 = severe limitation of physical activity due to dyspnea) and has a four-week recall period. A one-point change in the RDS score is considered clinically important [14].

The two-item PHQ-2 screens for depressive symptoms during a 2-week recall period and generates a summary score (scale 0‒6, 0 = no depressive symptoms, 6 = severe depressive symptoms). A PHQ-2 score of two or more points indicates depressive symptoms in CAD patients [19].

The generic HRQoL instrument 15D measures fifteen dimensions of health: “mobility”, “vision”, “hearing”,”breathing”, “sleeping”, “eating”, “speech”, “excretion”, “usual activities”, “mental function”, “discomfort and symptoms”, “depression”, “distress”, “vitality”, and “sexual activity” [7]. Each dimension question has five response options describing the present health of the patient. The single index score (15D score), representing the overall HRQoL on a 0–1 scale (1 = full health, 0 = being dead) and the dimension level values, reflecting the goodness of the levels relative to no problems on the dimension (= 1), and to being dead (= 0), are calculated from the health state descriptive system (questionnaire) by using a set of population-based preference or utility weights. Based on age, gender, and other patients’ responses, one to three missing 15D answers can be imputed using regression analysis [20]. A positive change of > 0.015 in the overall 15D score indicates a clinically important improvement [21].

### Statistical analysis

Statistical analysis was carried out by using the IBM SPSS statistical software (IBM SPSS, Inc., Chicago, IL, USA, version 25). The results are given as mean (standard deviation, SD), mean (95% confidence interval, CI), or percentages. The distribution of scores at the floor (worst possible scores) and the ceiling (best possible scores) of the instrument scales was explored. According to a previous work, floor, or ceiling effects of < 15% are considered acceptable in health status questionnaires [22]. High proportions of floor and ceiling scores prior to treatment may complicate the assessment of health benefit. Changes in instruments domain and total scores between the baseline and the 1-year follow-up measurement were examined with linear mixed model adjusting for the baseline value. To investigate whether the effect of a baseline score on the change in score differed between the treatment groups, a model with baseline scores and treatment group interaction was fitted. Statistically significant interactions are reported in the results. *P*-values < 0.05 were considered statistically significant.

To evaluate whether the 15D provides information included in the ICHOM standard set, we explored nonparametric Spearman correlations, suitable for ordinal and nonnormal data, between disease-specific instrument scores and SAQ-7 domain scores and the 15D at baseline, as well as the correlation of change in scores during 1-year follow-up. The opposite scales of RDS and PHQ-2 were reverse coded (by multiplying the scores by − 1) for the correlation analysis. Correlation coefficient values r < 0.3 were considered poor, values 0.3 ≤ r < 0.6 fair, values 0.6 ≤ r < 0.8 moderately strong, and values r ≥ 0.8 very strong [23]. Spearman correlation assumes a monotonic relationship between the investigated variables. Investigation of pairwise scatterplots did not implicate nonmonotonic relationship.

The baseline 15D dimension values, SAQ-PL, SAQ-AF, SAQ-QL, RDS, and PHQ-2 scores were included in an explanatory factor analysis to explore, to what extent there is common variability among these variables, and whether the interrelationships between the variables can be presented in a condensed way as fewer interpretable, underlying, or latent variables, i.e., factors. Similarly, the 1-year change in these observed variables were included in an explanatory factor analysis. First, principal components with an eigenvalue > 1 were extracted and then Varimax–rotated. In the interpretation of factors, attention was paid to loadings > 0.5, and especially to the highest loadings.

### Ethical considerations

The study was approved by the Research Ethics Committee of the Northern Savo Hospital District and registered with trial number 5101114. All study participants gave written consent, and decision to participate in this study did not affect their treatment.

## Results

The baseline characteristics of the 279 included study participants are shown in Additional file 1: Table S1. The proportion of PCI treated respondents was significantly higher in the group of respondents included in the study compared to those excluded.

### SAQ-7, RDS, PHQ-2 and the generic 15D at baseline and at 1-year follow-up

The mean baseline instrument scores, mean 1-year changes, and the proportions of floor and ceiling scores at baseline and at 1-year follow-up for each instrument are presented in Table 1.

**Table 1:** Mean scores, mean change, and estimated 1-year follow-up changes, and floor and ceiling scores

Instrument score	BL mean value (95% CI)	1 year follow-up change (95% CI)				Floor, n (%)		Ceiling, n (%)	
		Mean	OMT^a^	PCI^a^	CABG^a^	BL	1 yr	BL	1 yr
SAQ-7	63.3 (60.8–65.8)	16.4 (14.0–18.9)	11.4 (9.1–15.9)	17.6 (14.2–19.6)	30.2 (21.1–35.0)	1 (0.4)	1 (0.4)	18 (6.5)	63 (22.6)
SAQ-PL	76.5 (73.8–79.2)	5.1 (2.7–7.6)	3.7 (− 1.0 to 6.6)	5.0 (2.8–8.6)	10.0 (3.1–18.2)	2 (0.8)	2 (0.7)	66 (25.8)	92 (37.1)
SAQ-AF	65.6 (62.6–68.7)	17.8 (14.9–20.7)	12.4 (10.7–18.1)	19.7 (15.3–21.2)	27.9 (20.0–24.9)	3 (1.1)	2 (0.7)	38 (13.7)	125 (44.8)
SAQ-QL	48.8 (45.4–52.2)	25.7 (21.8–29.6)	18.0 (15.5–25.6)	27.3 (22.3–30.2)	47.4 (29.8–50.7)	13 (4.7)	3 (1.1)	25 (9.1)	107 (38.5)
RDS	1.9 (1.7–2.0)	0.5 (0.4–0.6)	0.2 (0.1–0.4)	0.6 (0.4–0.7)	1.0 (0.6–1.4)	27 (9.7)	19 (6.8)	42 (15.1)	83 (29.7)
PHQ-2	1.0 (0.8–1.1)	0.1 (0.0–0.3)	0.1 (− 0.2 to 0.2)	0.1 (0.0–0.3)	0.5 (0.0–0.9)	5 (1.8)	5 (1.8)	143 (51.3)	167 (59.9)
15D	0.818 (0.806–0.830)	0.024 (0.016–0.032)	0.005 (− 0.009 to 0.017)	0.028 (0.019–0.039)	0.074 (0.051–0.104)	1 (0.4)	1 (0.4)	3 (1.1)	3 (1.1)
15D dimension values									
Mobility	0.817 (0.797–0.837)	0.030 (0.010–0.049)	0.015 (− 0.015 to 0.045)	0.033 (0.009–0.057)	0.077 (0.014–0.140)	21 (7.5)	3 (1.1)	119 (42.7)	150 (53.8)
Vision	0.917 (0.899–0.934)	0.010 (− 0.010 to 0.029)	0.010 (− 0.016 to 0.037)	0.011 (− 0.011 to 0.032)	− 0.014 (− 0.068 to 0.041)	5 (1.8)	6 (2.2)	196 (70.3)	205 (73.5)
Hearing	0.895 (0.875–0.915)	0.005 (− 0.011 to 0.021)	− 0.003 (− 0.027 to 0.021)	0.008 (− 0.012 to 0.027)	0.018 (− 0.032 to 0.067)	1 (0.4)	21 (7.5)	189 (67.7)	191 (68.5)
Breathing	0.657 (0.630–0.684)	0.105 (0.079–0.130)	0.071 (0.036–0.107)	0.104 (0.075–0.133)	0.241 (0.168–0.315)	2 (0.7)	1 (0.4)	60 (21.5)	110 (39.4)
Sleeping	0.760 (0.736–0.785)	0.019 (− 0.001 to 0.039)	0.003 (− 0.026 to 0.032)	0.020 (− 0.003 to 0.043)	0.081 (0.023–0.140)	30 (7.2)	1 (0.4)	86 (30.8)	89 (31.9)
Eating	0.987 (0.980–0.995)	0.006 (0.000–0.013)	0.003 (− 0.005 to 0.010)	0.007 (0.001–0.013)	0.013 (− 0.003 to 0.028)	10 (3.6)	5 (1.8)	269 (96.4)	374 (89.2)
Speech	0.959 (0.946–0.972)	− 0.005 (− 0.017 to 0.007)	− 0.020 (− 0.038 to [− 0.002])	0.002 (− 0.013 to 0.016)	0.006 (− 0.030 to 0.043)	2 (0.7)	2 (0.7)	242 (86.7)	237 (84.9)
Excretion	0.878 (0.815–0.860)	− 0.013 (− 0.037 to 0.010)	− 0.048 (− 0.082 to [− 0.014])	0.007 (− 0.020 to 0.034)	0.023 (− 0.065 to 0.072)	1 (0.4)	20 (7.2)	151 (54.1)	142 (50.9)
Usual activities	0.758 (0.732–0.783)	0.030 (0.007–0.053)	− 0.011 (− 0.044 to 0.023)	0.040 (0.013–0.067)	0.130 (0.062–0.199)	6 (2.2)	5 (1.8)	97 (34.8)	116 (41.6)
Mental functions	0.876 (0.855–0.897)	− 0.013 (− 0.032 to 0.005)	− 0.040 (− 0.069 to [− 0.011])	0.003 (− 0.020 to 0.027)	0.003 (− 0.056 to 0.062)	1 (0.4)	1 (0.4)	185 (66.3)	179 (64.2)
Discomfort and symptoms	0.671 (0.645–0.697)	0.051 (0.026–0.075)	0.025 (− 0.011 to 0.060)	0.056 (0.028–0.085)	0.140 (0.066–0.215)	3 (1.1)	14 (5.0)	49 (17.6)	77 (27.6)
Depression	0.890 (0.873–0.907)	0.010 (− 0.005 to 0.025)	− 0.007 (− 0.030 to 0.015)	0.013 (− 0.004 to 0.031)	0.069 (0.024–0.115)	1 (0.4)	3 (1.1)	162 (58.1)	177 (63.4)
Distress	0.870 (0.851–0.888)	0.024 (0.006–0.042)	0.011 (− 0.014 to 0.036)	0.025 (0.005–0.045)	0.072 (0.020–0.123)	1 (0.4)	10 (3.6)	159 (57.0)	180 (64.5)
Vitality	0.734 (0.715–0.753)	0.054 (0.035–0.073)	0.026 (− 0.002 to 0.053)	0.058 (0.036–0.081)	0.145 (0.088–0.202)	5 (1.8)	8 (2.9)	42 (15.1)	76 (27.2)
Sexual activity	0.671 (0.638–0.705)	0.026 (0.001–0.052)	− 0.001 (− 0.039 to 0.038)	0.026 (− 0.005 to 0.057)	0.148 (0.068–0.227)	18 (6.8)	20 (7.2)	92 (33.0)	102 (36.6)

BL, Baseline; CABG, coronary artery bypass grafting; CI, confidence interval; OMT, optimal medical therapy; PCI, percutaneous coronary intervention; PHQ-2, two-item Patient Health Questionnaire; RDS, Rose Dyspnea Scale; SAQ-AF, Seattle Angina Questionnaire Angina Frequency; SAQ-PL, Seattle Angina Physical Limitation; SAQ-QL, Seattle Angina Quality of Life; SAQ-7, Seattle Angina Questionnaire short-form; SD, Standard Deviation; 1 yr, one-year; 15D, 15-dimensional instrument

^a^Adjusted for baseline scores

During the 1-year follow-up, the mean change in SAQ-7 was 16.4 (CI 14.0–18.9), and the estimated changes in the treatment groups were 12.5 (CI 9.1–15.9) for OMT, 16.9 (14.2–19.6) for PCI, and 28.0 (CI 21.1–35.0) for CABG. The mean changes in the domain scores were 5.1 (CI 2.6–7.6) for SAQ-PL, 17.8 (CI 14.9–20.7) for SAQ-AF, and 25.7 (CI 21.8–29.6) for SAQ-QL. The estimated mean changes in the domain scores within treatment groups are presented in Table 1. The proportion of ceiling scores of SAQ-7 increased from 6.5% at baseline to 22.6% at 1 year. The proportion of ceiling domain scores increased significantly from 25.8% (n = 66) at baseline to 37.1% (n = 92) at 1 year for SAQ-PL, 13.7% (n = 38) at baseline to 44.8% (n = 125) at 1 year for SAQ-AF, and from 9.1% (n = 25) at baseline to 38.5% (n = 107) for SAQ-QL.

The 1-year mean change in RDS score was 0.5 (CI 0.4–0.6) and estimated changes in the treatment groups were 0.2 (CI 0.1–0.4) for OMT, 0.6 (CI 0.4–0.7) for PCI and 1.0 (0.7–1.4) for CABG. The proportion of patients without dyspnea (ceiling scores) nearly doubled from 15.1% (n = 42) at baseline to 29.7% (n = 83) at 1 year.

The mean change in PHQ-2 score was 0.1 (CI 0.0–0.3) during the 1-year follow-up. The estimated mean change at 1-year was 0.1 (CI − 0.2 to 0.2) for OMT, 0.1 (CI 0.0–0.3) for PCI, and 0.5 (CI 0.0–0.9) for CABG. The proportion of ceiling PHQ-2 scores showed slight, but not significant, change from 51.3% (n = 143) at baseline to 59.9% (n = 167) at 1 year.

The 1-year change in mean overall 15D score was 0.024 (CI 0.016–0.032) and the estimated mean changes were 0.005 (CI − 0.009 to 0.017) for OMT, 0.028 (CI 0.019–0.039) for PCI, and 0.074 (CI 0.051–0.104) for CABG. The association between baseline scores and 1-year change in the 15D dimensions “eating”, “speech”, “discomfort and symptoms” and “sexual activity” were different in the treatment groups (*p* for baseline dimension and treatment group interaction < 0.05). The estimated differences within the groups are shown at Table 1.

The proportion of overall 15D scores at the ceiling did not differ between baseline (1.1%) and 1-year follow-up (1.1%). However, the proportion of 15D dimension values at the ceiling changed as follows from baseline to 1-year: “mobility” from 42.7 to 53.8%, “breathing” from 21.5 to 39.4%, “usual activities” from 34.8 to 41.6%, “discomfort and symptoms” from 17.6 to 27.6%, “distress” from 57.0 to 64.5%, “vitality” from 15.1 to 27.2%, and”sexual activity” from 33.0 to 36.6%.

### Correlation between the instrument scores of SAQ-7, RDS, PHQ-2 and the 15D score and 15D dimension values at baseline and at 1-year follow-up

**Fig. 1:**
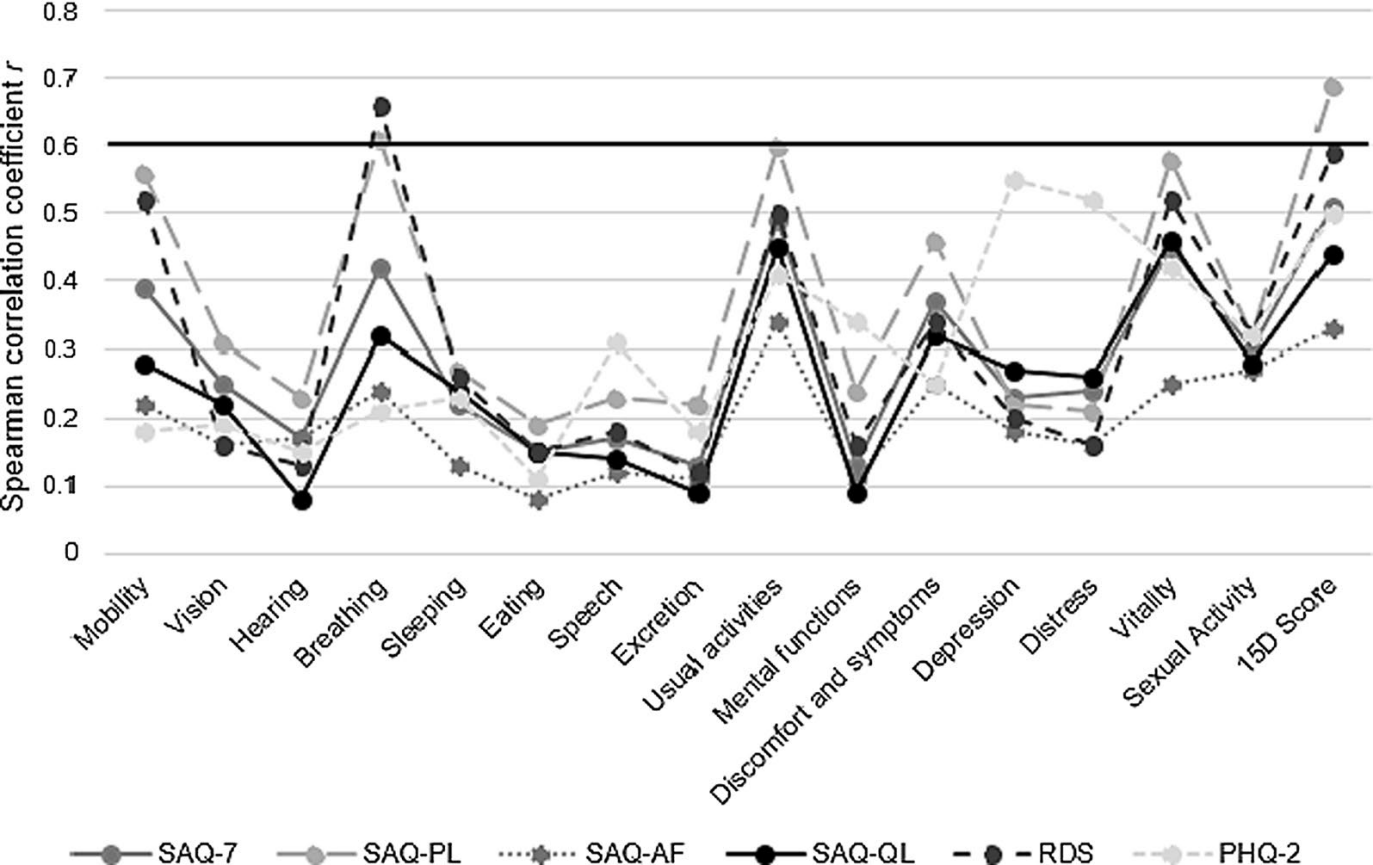
Spearman correlations between the overall 15D score and dimension values and the Seattle Angina Questionnaire short-form (SAQ-7), SAQ Physical Limitation (SAQ-PL), SAQ Angina Frequency (SAQ-AF), SAQ Quality of Life (SAQ-QL), Rose Dyspnea Scale (RDS) and two-item Patient Health Questionnaire (PHQ-2) at baseline

Correlation coefficients of the 15D instrument scores and the ICHOM standard set instruments at baseline are presented in Fig. 1 and Additional file 1: Table S2 and at 1-year follow-up in Additional file 1: Table S2. At baseline, the overall 15D score and SAQ-PL (r = 0.69), the 15D dimension value of “breathing” and SAQ-PL (r = 0.61), the 15D dimension value of “usual activities” and SAQ-PL (r = 0.60), as well as the 15D dimension value of “breathing” and RDS (r = 0.66) showed moderately strong correlation (0.6 ≤ r < 0.8). The other correlations were fair (0.3 ≤ r < 0.6) or poor (r < 0.3). At 1-year follow-up, correlations were fairly similar to those observed at baseline (Additional file 1: Table S2).

The factor analysis of baseline scores identified five factors (Table 2) that explained 61.0% of the total variance. The 15D dimension of “breathing” followed by RDS score, SAQ-PL, and the dimensions of “mobility”, “vitality”, and “usual activities” had high loadings on Factor 1. Based on the variables loading highly onto Factor 1, it could be interpreted to reflect “Breathing-related physical activity”, explaining 19.2% of the total variance.

**Table 2:** Rotated component matrix of the 15D dimension values and instrument scores

Item at baseline	Factor (% of variance explained)				
	1 (32.9%)	2 (9.8%)	3 (7.1%)	4 (5.8%)	5 (5.4%)
SAQ-PL	**0.70**	0.12	0.29	0.24	0.18
SAQ-AF	0.19	0.08	**0.87**	0.02	0.11
SAQ-QL	0.35	0.23	**0.79**	0.07	0.01
RDS	** − 0.81**	− 0.11	− 0.16	− 0.05	0.01
PHQ-2	− 0.21	− **0.62**	− 0.20	− 0.04	− 0.22
15D mobility	**0.74**	0.01	0.05	0.18	0.12
15D vision	0.12	0.16	0.14	0.36	0.47
15D hearing	0.13	− 0.14	0.16	0.21	**0.69**
15D breathing	**0.83**	0.03	0.07	0.13	0.09
15D sleeping	0.25	0.37	− 0.02	**0.61**	− 0.14
15D eating	0.14	− 0.07	0.05	**0.65**	0.18
15D speech	0.12	0.46	− 0.12	0.16	**0.53**
15D excretion	0.05	0.14	0.01	**0.67**	0.17
15D usual activities	**0.60**	0.33	0.29	0.03	0.34
15D mental functions	0.13	**0.54**	− 0.14	− 0.06	**0.50**
15D discomfort and symptoms	0.37	0.28	0.28	0.35	0.07
15D depression	0.13	**0.81**	0.12	0.15	− 0.06
15D distress	0.02	**0.82**	0.15	0.15	0.06
15D vitality	**0.62**	0.46	0.18	0.20	0.06
15D sexual activity	0.45	0.31	0.08	− 0.16	0.36

The highest loadings are bolded

PHQ-2, two-item Patient Health Questionnaire; RDS, Rose Dyspnea Scale; SAQ-AF, Seattle Angina Questionnaire Angina Frequency; SAQ-PL, Seattle Angina Questionnaire Physical Limitation; SAQ-QL, Seattle Angina Questionnaire Quality of Life; SAQ-7, Seattle Angina Questionnaire short-form; 15D, 15-dimensional instrument

The 15D dimension of “distress” followed by “depression”, PHQ-2 score, and the 15D dimension of “mental functions” had high loadings on Factor 2. For this, Factor 2 could be interpreted to reflect “Mental health”, explaining 15.0% of the total variance.

The variables SAQ-AF and SAQ-QL had high loadings on Factor 3. Based on these variables, Factor 3 could be interpreted to reflect disease-specific, *i.e*., “Angina-related quality of life”, explaining 9.3% of the total variance. Factors 4 and 5 were 15D-specific factors, reflecting health problems CAD patients may have, be they CAD-related or not.

Correlations between the 1-year changes in the scores of the 15D variables and those of the ICHOM standard set were only fair at best (Table 3). The highest correlations (r = 0.40‒0.42) were observed between the overall 15D score and the RDS, PHQ-2, SAQ-7, SAQ-PL, and SAQ-QL.

**Table 3:** Spearman correlations between 1-year changes in the 15D variables and instrument scores

Item	SAQ-7	SAQ-PL	SAQ-AF	SAQ-QL	RDS	PHQ-2
Mobility	0.25	0.31	0.18	0.23	0.20	0.15
Vision	0.03	0.05	0.04	− 0.01	0.03	0.03
Hearing	0.06	0.04	0.06	0.05	0.08	0.01
Breathing	0.31	0.25	0.23	0.31	0.42	0.20
Sleeping	0.10	0.01	0.10	0.14	0.12	0.03
Eating	0.09	0.13	0.06	0.08	0.04	0.06
Speech	0.02	0.04	− 0.01	− 0.04	0.13	0.07
Excretion	0.07	0.10	0.10	0.05	− 0.05	0.16
Usual activities	0.36	0.36	0.24	0.35	0.23	0.31
Mental functions	0.03	0.02	0.00	0.05	0.14	0.15
Discomfort and symptoms	0.24	0.17	0.23	0.21	0.13	0.16
Depression	0.21	0.22	0.20	0.22	0.20	0.38
Distress	0.21	0.13	0.18	0.22	0.22	0.34
Vitality	0.35	0.31	0.22	0.36	0.29	0.31
Sexual activity	0.29	0.26	0.17	0.26	0.26	0.30
Overall 15D score	0.42	0.40	0.33	0.41	0.40	0.41

The Spearman correlation coefficient values r < 0.3 were considered poor, values 0.3 ≤ r < 0.6 fair, values 0.6 ≤ r < 0.8 moderately strong, and values r ≥ 0.8 strong

PHQ-2, two-item Patient Health Questionnaire; RDS, Rose Dyspnea Scale; SAQ-AF, Seattle Angina Questionnaire Angina Frequency; SAQ-PL, Seattle Angina Questionnaire Physical Limitation; SAQ-QL, Seattle Angina Questionnaire Quality of Life; SAQ-7, Seattle Angina Questionnaire short-form; 15D, 15-dimensional instrument

The factor analysis based on these change variables identified 7 factors (Table 4) that explained 58.2% of the total variance. The interpretation of Factors 1, explaining 20.5% of the total variance, and Factor 2, explaining 7.5% of the total variance, is similar to Factors 1 (“Breathing-related physical activity”) and 2 (“Mental health”) based on baseline scores.

**Table 4:** Rotated component matrix of the 1-year changes in 15D dimension values and instrument scores

One-year change in item	Factor (% of variance explained)						
	1 (20.6%)	2 (7.5%)	3 (7.5%)	4 (6.2%)	5 (5.8%)	6 (5.5%)	7 (5.2%)
SAQ-PL	**0.65**	− 0.03	− 0.10	0.18	0.21	0.07	0.04
SAQ-AF	0.33	0.40	− 0.11	0.25	**0.57**	− 0.18	0.09
SAQ-QL	**0.55**	0.44	− 0.12	0.11	0.35	− 0.23	0.09
RDS	**0.64**	− 0.17	− 0.22	0.25	− 0.11	0.04	0.17
PHQ-2	− 0.19	** − 0.65**	0.05	− 0.16	0.04	0.10	− 0.02
15D mobility	**0.52**	0.00	− 0.20	− 0.17	0.07	− 0.10	0.24
15D vision	0.09	0.05	0.01	0.11	− 0.08	**0.80**	0.05
15D hearing	0.05	− 0.16	0.23	− 0.02	**0.74**	0.03	0.03
15D breathing	**0.56**	0.14	0.24	− 0.17	0.16	0.19	0.12
15D sleeping	0.26	− 0.05	0.36	0.33	− 0.35	− 0.42	0.14
15D eating	0.06	− 0.02	− 0.05	− 0.10	0.10	0.13	**0.85**
15D speech	0.02	− 0.06	**0.57**	0.01	0.10	0.44	0.15
15D excretion	− 0.03	0.08	0.03	**0.75**	0.07	0.10	− 0.02
15D usual activities	**0.62**	0.13	− 0.05	0.44	0.01	0.01	− 0.09
15D mental functions	− 0.02	0.10	**0.78**	0.02	0.09	− 0.11	− 0.08
15D discomfort and symptoms	0.15	0.25	0.17	0.35	− 0.14	− 0.19	0.49
15D depression	0.11	**0.66**	0.22	0.07	− 0.03	0.25	− 0.08
15D distress	0.10	**0.75**	0.01	− 0.08	− 0.02	− 0.07	0.09
15D vitality	**0.60**	0.21	0.12	0.19	− 0.11	− 0.08	0.12
15D sexual activity	**0.60**	0.20	− 0.01	0.04	− 0.25	0.11	0.03

The highest loadings are bolded

PHQ-2, two-item Patient Health Questionnaire; RDS, Rose Dyspnea Scale; SAQ-AF, Seattle Angina Questionnaire Angina Frequency; SAQ-PL, Seattle Angina Questionnaire Physical Limitation; SAQ-QL, Seattle Angina Questionnaire Quality of Life; SAQ-7, Seattle Angina Questionnaire short-form; 15D, 15-dimensional instrument

The rest of the factors were mainly 15D-specific, but quite difficult to interpret, although again they seem to reflect health problems CAD patient may have, be they CAD-related or not.

## Discussion

Our study is the first to explore the correlation of scores and dimension values generated by the generic HRQoL instrument 15D with the scores of instruments included in the ICHOM standard set for treatment outcome measurement in CAD. It demonstrated improvement in all four instrument scores during 1-year follow-up.

The 15D dimension value of “breathing” and the RDS score showed moderately strong correlation at baseline. Consistently, in the factor analysis of baseline scores, the 15D dimension of “breathing” and the RDS score had strongest loadings to factor 1 reflecting “Breathing-related physical activity”. Consistent with our results, Mazur et al. previously demonstrated strong correlation between the 15D and dyspnea assessed with the disease-specific Airways questionnaire 20 in COPD patients [24].

Previously, the 15D dimension of “depression” demonstrated strong to very strong correlation with the Beck Depression Inventory in patients with depressive disorders, both at baseline and at 5-year follow-up [25]. Correspondingly, in our study the factor analysis of baseline scores revealed significant loadings of the 15D dimension of “depression”, and the PHQ-2 score on Factor 2 named “Mental health”. Furthermore, factor analysis of 1-year changes in scores demonstrated the importance of the mental health factor in CAD, as the 1-year change in “distress”, “depression” and the PHQ-2 score were significantly loaded on Factor 2.

Anxiety is recognized as a comorbidity in CAD [26, 27], and was recently, in a large study, found to predict cardiac readmission [28]. Unlike any of the instruments included in the ICHOM standard set, the 15D instrument measures anxiety (dimension of “distress”) in addition to “depression”. We found fair correlation between the baseline 15D dimension value of “distress” and the PHQ-2 score. Moreover, the importance of “distress” was supported by significant loadings on the factor of “Mental health” both at baseline and on factor based on 1-year change in scores.

Our study found fair correlation between the overall 15D score and the SAQ-PL at baseline, and furthermore, the factor analysis of baseline values showed that the 15D dimensions reflecting physical health together with “breathing” and the SAQ-PL score loaded highly on the same factor named “Breathing-related physical activity”.

The factor analysis based on 1-year changes in scores revealed that the change in the disease-specific quality of life measured with SAQ-QL had moved from the baseline factor of “Angina-related quality of life” to the change factor of “Breathing-related physical activity”. Even though conclusions should not be made based solely on the explanatory factor analysis of a rather small data, this may indicate that a variation in the change of generic captures the variation in the changes in breathing and physical symptoms more strongly than it captures the variation in change of anginal symptoms.

However, electively treated CAD patients may have adapted their physical activity level prior to treatment to avoid anginal symptoms, and thus scored better in SAQ-AF at baseline. Consequently, they may have perceived treatment benefit mainly as improved physical activity during the follow-up. The substantially larger proportion of ceiling SAQ-AF and RDS scores observed at 1-year follow-up may also reflect health gain. It may also be explained by the fact that only respondents with instrument scores at baseline and at 1-year follow-up were included in the study and thus, healthier respondents may be represented.

Previous work has demonstrated that the four-week recall period of SAQ-7 is reliable compared with daily self-reporting of angina [29]. Although 15D is a validated instrument in patients with chronic pain [8, 30], it did not correlate, or correlated only poorly, with the disease-specific angina pain frequency measured with the SAQ-AF. Moreover, the factor analysis based on baseline scores and 1-year change scores confirmed that the SAQ-AF and the 15D dimension of “discomfort and symptoms” did not load on the same factor.

This lack of correlation might be explained by the fact that the 15D records present symptoms. Thus, the absence of anginal symptoms at the time of responding to the 15D questionnaire, may explain the modest correlation between the “discomfort and symptoms” dimension and the SAQ-AF score. Additionally, the 15D dimension “discomfort and symptoms” is not limited solely to pain, as it includes other types of physical discomfort and symptoms such as itching and nausea. Consequently, the dimension is not directly comparable with the SAQ-AF that measures anginal frequency. However, considering the importance of capturing anginal symptoms in CAD, this may call for combining this disease-specific variable to a generic HRQoL instrument, like the 15D, in CAD patients.

To achieve better reliability in the correlation analysis, the study was limited to those who responded to all four questionnaires at both baseline and at 1-year follow-up. It is possible that those with worse health may not have responded to all four questionnaires at 1 year which is a limitation of the study [31].

To the best of our knowledge, this is the first study to compare the performance of the 15D with the instruments included in the ICHOM standard set in CAD patients who have received OMT or undergone PCI, or CABG in a routine care setting. Another strength of our study is the utilization of the 15D instrument to measure generic HRQoL as it has been found to have higher discriminatory power and better validity in the disease area of heart disease than some other generic instruments [10, 11, 32, 33].

## Conclusions

The 15D instrument partially captured dyspnea, physical limitation, and depression measured by the instruments included in the ICHOM standard set for CAD. Still, as implied by the modest to moderately strong correlations, the SAQ-7, RDS and PHQ-2 capture slightly different information than the 15D. However, the 15D dimension of “discomfort and symptoms” showed only modest correlation with angina frequency measured by the SAQ-AF, which indicates that to detect angina, the 15D instrument should be supplemented with a disease-specific instrument.

## Supplementary Information

**Additional file 1**.** Table S1**. Baseline clinical characteristics.** Table S2**. Correlation between the 15D dimension values and instrument scores of instruments scores of Seattle Angina Questionnaire short-form (SAQ-7), SAQ Physical Limitation (SAQ-PL), SAQ Angina Frequency (SAQ-AF), SAQ Quality of Life (SAQ-QL), Rose Dyspnea Scale (RDS), and two-item Patient Health Questionnaire (PHQ-2) at baseline (BL) and at one-year (1yr) follow-up. The moderately high correlations are bolded.

## Data Availability

The datasets generated and analyzed during the current study are not available due to participants’ privacy but are available from the corresponding author on reasonable request.

## Abbreviations

15D: Fifteen Dimensional health-related quality of life instrument
AQoL: Assessment of Quality of Life instrument
CAD: Coronary artery disease
CABG: Coronary artery bypass grafting
CI: Confidence interval
COPD: Chronic obstructive pulmonary disease
EQ-5D: Five Dimensional EuroQol health-related quality of life instrument
HRQoL: Health-related quality of life
HUI3: Health Utilities Index mark 3 instrument
ICHOM: International Consortium for Health Outcomes Measurement
OMT: Optimal medical therapy
PCI: Percutaneous coronary intervention
PHQ-2: Two-item Patient Health Questionnaire
PRO: Patient-reported outcome
QALY: Quality-adjusted life years
RDS: Rose Dyspnea Scale
SAQ-7: Seattle Angina Questionnaire short-form
SAQ-AF: Seattle Angina Questionnaire domain measuring anginal frequency
SAQ-PL: Seattle Angina Questionnaire domain measuring physical limitation
SAQ-QL: Seattle Angina Questionnaire domain measuring quality of life
SD: Standard deviation
SF-6D: Short-Form Six-Dimension health index

## References

[1] Edwards P, Roberts I, Sandercock P, Frost C (2004). Follow-up by mail in clinical trials: does questionnaire length matter?. Control Clin Trials.

[2] Spertus JA, McDonell M, Woodman CL, Fihn SD (2000). Association between depression and worse disease-specific functional status in outpatients with coronary artery disease. Am Heart J.

[3] Ulvik B, Bjelland I, Hanestad BR, Omenaas E, Wentzel-Larsen T, Nygard O (2008). Comparison of the Short Form 36 and the Hospital Anxiety and Depression Scale measuring emotional distress in patients admitted for elective coronary angiography. Heart Lung.

[4] Moock J, Kohlmann T (2008). Comparing preference-based quality-of-life measures: results from rehabilitation patients with musculoskeletal, cardiovascular, or psychosomatic disorders. Qual Life Res.

[5] Schweikert B, Hahmann H, Leidl R (2006). Validation of the EuroQol questionnaire in cardiac rehabilitation. Heart.

[6] De Smedt D, Clays E, Doyle F, Kotseva K, Prugger C, Pajak A (2013). Validity and reliability of three commonly used quality of life measures in a large European population of coronary heart disease patients. Int J Cardiol.

[7] 15D instruments. http://www.15d-instrument.net/15d. Accessed 3 Feb 2021.

[8] Vartiainen P, Mäntyselkä P, Heiskanen T, Hagelberg N, Mustola S, Forssell H (2017). Validation of EQ-5D and 15D in the assessment of health-related quality of life in chronic pain. Pain.

[9] Hawthorne G, Richardson J, Day NA (2001). A comparison of the Assessment of Quality of Life (AQoL) with four other generic utility instruments. Ann Med.

[10] Heiskanen J, Tolppanen A-M, Roine RP, Hartikainen J, Hippeläinen M, Miettinen H (2016). Comparison of EQ-5D and 15D instruments for assessing the health-related quality of life in cardiac surgery patients. Eur Heart J Qual Care Clin Outcomes.

[11] Richardson J, Iezzi A, Khan MA, Chen G, Maxwell A (2016). Measuring the sensitivity and construct validity of 6 utility instruments in 7 disease areas. Med Decis Mak.

[12] McNamara RL, Spatz ES, Kelley TA, Stowell CJ, Beltrame J, Heidenreich P (2015). Standardized Outcome Measurement for Patients with Coronary Artery Disease: Consensus from the International Consortium for Health Outcomes Measurement (ICHOM). J Am Heart Assoc.

[13] Chan PS, Jones PG, Arnold SA, Spertus JA (2014). Development and validation of a short version of the Seattle angina questionnaire. Circ Cardiovasc Qual Outcomes.

[14] Rose GA, Blackburn H, Gillum RF, Prineas RJ. Cardiovascular survey methods. World Health Organization. Monograph series; 1982; no. 56, p. 1–888. 4972212

[15] Kroenke K, Spitzer RL, Williams JBW (2003). The Patient Health Questionnaire-2: validity of a two-item depression screener. Med Care.

[16] Löwe B, Kroenke K, Gräfe K (2005). Detecting, and monitoring depression with a two-item questionnaire (PHQ-2). J Psychosom Res.

[17] Bruckel J, Wagle N, O'Brien C, Elias J, McKenna S, Meyers P (2015). Feasibility of a tablet computer system to collect patient-reported symptom severity in patients undergoing diagnostic coronary angiography. Crit Pathw Cardiol.

[18] Yang JX, Stevenson MJ, Valsdottir L, Ho K, Spertus JA, Yeh RW (2020). Association between procedure appropriateness and patient-reported outcomes after percutaneous coronary intervention. Heart.

[19] Thombs BD, Ziegelstein RC, Whooley MA (2008). Optimizing detection of major depression among patients with coronary artery disease using the patient health questionnaire: data from the Heart and Soul study. J Gen Intern Med.

[20] Sintonen H (2001). The 15D instrument of health-related quality of life: properties and applications. Ann Med.

[21] Alanne S, Roine RP, Räsänen P, Vainiola T, Sintonen H (2015). Estimating the minimum important change in the 15D scores. Qual Life Res.

[22] McHorney CA, Tarlov AR (1995). Individual-patient monitoring in clinical practice: are available health status surveys adequate?. Qual Life Res.

[23] Chan YH (2003). Biostatistics 104: correlational analysis. Singap Med J.

[24] Mazur W, Kupiainen H, Pitkäniemi J, Kilpeläinen M, Sintonen H, Lindqvist A (2011). Comparison between the disease-specific Airways Questionnaire 20 and the generic 15D instruments in COPD. Health Qual Life Outcomes.

[25] Riihimäki K, Sintonen H, Vuorilehto M, Jylhä P, Saarni S, Isometsä E (2016). Health-related quality of life of primary care patients with depressive disorders. Eur Psychiatr.

[26] Jespersen L, Abildstrøm SZ, Hvelplund A, Prescott E (2013). Persistent angina: highly prevalent and associated with long-term anxiety, depression, low physical functioning, and quality of life in stable angina pectoris. Clin Res Cardiol.

[27] Korbmacher B, Ulbrich S, Dalyanoglu H, Lichtenberg A, Schipke JD, Franz M (2013). Perioperative and long-term development of anxiety and depression in CABG patients. Thorac Cardiovasc Surg.

[28] Vámosi M, Lauberg A, Borregaard B, Christensen AV, Thrysoee L, Rasmussen TB (2020). Patient-reported outcomes predict high readmission rates among patients with cardiac diagnoses. Findings from the DenHeart study. Int J Cardiol.

[29] Arnold SV, Kosiborod M, Li Y, Jones PG, Yue P, Belardinelli L (2014). Comparison of the Seattle angina questionnaire with daily angina diary in the TERISA clinical trial. Circul Cardiovasc Qual Outcomes.

[30] Vartiainen P, Heiskanen T, Sintonen H, Roine RP, Kalso E (2016). Health-related quality of life and burden of disease in chronic pain measured with the 15D instrument. Pain.

[31] Saarni SI, Härkänen T, Sintonen H, Suvisaari J, Koskinen S, Aromaa A (2006). The impact of 29 chronic conditions in health-related quality of life: a general population survey in Finland using 15D and EQ-5D. Qual Life Res.

[32] Vainiola T, Pettilä V, Roine RP, Räsänen P, Rissanen AM, Sintonen H (2010). Comparison of two utility instruments, the EQ-5D and the 15D, in the critical care setting. Intensive Care Med.

[33] Oinasmaa S, Heiskanen J, Hartikainen J, Hippeläinen M, Miettinen H, Martikainen J (2018). Does routinely collected patient-reported outcome data represent the actual case-mix of elective coronary revascularization patients?. Eur Heart J Qual Care Clin Outcomes.

