# Supplementary material for: Patient-reported outcomes in coronary artery disease: the relationship between the standard, disease-specific set by the International Consortium for Health Outcomes Measurement (ICHOM) and the generic health-related quality of life instrument 15D
Source: Health Qual Life Outcomes. 2021 Aug 28;19:206. doi: 10.1186/s12955-021-01841-6 (PMC8401180; doi:10.1186/s12955-021-01841-6)
Supplement: Supplementary file 1 — Additional file 1. Table S1. Baseline clinical characteristics. Table S2. Correlation between the 15D dimension values and instrument scores of instruments scores of Seattle Angina Questionnaire short-form (SAQ-7), SAQ Physical Limitation (SAQ-PL), SAQ Angina Frequency (SAQ-AF), SAQ Quality of Life (SAQ-QL), Rose Dyspnea Scale (RDS), and two-item Patient Health Questionnaire (PHQ-2) at baseline (BL) and at one-year (1yr) follow-up. The moderately high correlations are bolded. [file 12955_2021_1841_MOESM1_ESM.docx]

**Additional file 1: Table S1.** Baseline clinical characteristics.

| **Characteristics** | **Respondents included in analysis**  (n= 279) | **Excluded respondents**  (n=118) | **p-value** |
| --- | --- | --- | --- |
| **Age, mean (SD)** | 69.2 (9.2) | 70.4 (8.9) | 0.216 |
| **Sex, men** | 186 (66.7) | 67 (56.8) | 0.061 |
| **Previous CAD** | 210 (75.3) | 85 (72.0) | 0.501 |
| **Previous MI** | 54 (19.4) | 29 (24.6) | 0.243 |
| **Previous PCI** | 103 (36.9) | 60 (50.8) | 0.010 |
| **Previous CABG** | 41 (14.7) | 19 (16.1) | 0.721 |
| **Hypertension** | 227 (81.7) | 100 (86.2) | 0.274 |
| **Hyperlipidemia** | 265 (95.3) | 111 (94.9) | 0.848 |
| **Diabetes mellitus** | 70 (25.1) | 39 (33.1) | 0.105 |
| **Obesity** | 90 (33.0) | 40 (33.9) | 0.858 |
| **Current smoker** | 30 (10.8) | 15 (12.7) | 0.575 |
| **COPD/asthma** | 47 (16.8) | 22 (18.6) | 0.186 |
| **Renal failure** | 55 (19.8) | 29 (25.0) | 0.250 |
| **PAD** | 16 (5.7) | 12 (10.2) | 0.115 |
| **CCS grading** |  |  | 0.313 |
| **Class I** | 25 (9.1) | 7 (6.0) |  |
| **Class II** | 162 (58.7) | 71 (60.7) |  |
| **Class III** | 86 (31.2) | 35 (29.9) |  |
| **Class IV** | 3 (1.1) | 4 (3.4) |  |
| **LVEF < 50%** | 22 (10.0) | 6 (7.5) | 0.505 |
| **Diseased vessels** |  |  | 0.894 |
| **1-VD** | 85 (30.7) | 40 (33.9) |  |
| **2-VD** | 76 (27.4) | 31 (26.3) |  |
| **3-VD** | 88 (31.8) | 34 (28.8) |  |
| **No significant stenoses** | 28 (10.1) | 13 (11.0) |  |
| **Left main stenosis** | 24 (8.7) | 12 (10.2) | 0.635 |
| **Treatment arm** |  |  | 0.058 |
| **OMT** | 100 (35.8) | 53 (44.9) |  |
| **PCI** | 155 (55.6) | 59 (50.0) |  |
| **CABG** | 24 (8.6) | 6 (5.1) |  |

Results are expressed as the mean (SD) or as n (%). P-values <0.05 were considered statistically significant.

Abbreviations: CABG, coronary artery bypass grafting; CAD, coronary artery disease; CCS Canadian Cardiovascular Society; COPD, chronic obstructive pulmonary disease; LVEF, left ventricular ejection fraction; MI, myocardial infarction; OMT, optimal medical therapy; PAD, peripheral artery disease; PCI, percutaneous coronary intervention; SD standard deviation.

**Additional file 1: Table S2.** Correlation between 15D variables and instruments scores at baseline and at one-year follow-up.

| **15D dimension** | **SAQ-7** | | **SAQ-PL** | | **SAQ-AF** | | **SAQ-QL** | | **RDS** | | **PHQ-2** | |
| --- | --- | --- | --- | --- | --- | --- | --- | --- | --- | --- | --- | --- |
|  | **BL** | **1yr** | **BL** | **1yr** | **BL** | **1yr** | **BL** | **1yr** | **BL** | **1yr** | **BL** | **1yr** |
| **Mobility** | 0.38 | 0.51 | 0.56 | **0.60** | 0.22 | 0.35 | 0.28 | 0.42 | 0.52 | 0.56 | 0.18 | 0.40 |
| **Vision** | 0.25 | 0.17 | 0.31 | 0.23 | 0.16 | 0.14 | 0.22 | 0.09 | 0.16 | 0.18 | 0.19 | 0.23 |
| **Hearing** | 0.17 | 0.11 | 0.24 | 0.25 | 0.17 | 0.05 | 0.08 | 0.04 | 0.13 | 0.14 | 0.15 | 0.13 |
| **Breathing** | 0.42 | 0.59 | **0.61** | 0.59 | 0.24 | 0.49 | 0.32 | 0.49 | **0.66** | **0.75** | 0.21 | 0.38 |
| **Sleeping** | 0.22 | 0.32 | 0.27 | 0.29 | 0.13 | 0.30 | 0.24 | 0.28 | 0.26 | 0.34 | 0.23 | 0.32 |
| **Eating** | 0.15 | 0.08 | 0.20 | 0.12 | 0.08 | 0.05 | 0.15 | 0.06 | 0.15 | 0.09 | 0.11 | 0.16 |
| **Speech** | 0.17 | 0.18 | 0.23 | 0.21 | 0.12 | 0.18 | 0.14 | 0.12 | 0.18 | 0.12 | 0.31 | 0.25 |
| **Excretion** | 0.14 | 0.21 | 0.21 | 0.27 | 0.11 | 0.24 | 0.09 | 0.16 | 0.12 | 0.24 | 0.18 | 0.23 |
| **Usual activities** | 0.49 | 0.54 | 0.60 | **0.64** | 0.34 | 0.40 | 0.45 | 0.47 | 0.50 | 0.54 | 0.41 | **0.64** |
| **Mental functions** | 0.13 | 0.17 | 0.24 | 0.21 | 0.11 | 0.15 | 0.09 | 0.12 | 0.16 | 0.16 | 0.34 | 0.28 |
| **Discomfort and symptoms** | 0.37 | 0.38 | 0.45 | 0.29 | 0.25 | 0.33 | 0.32 | 0.30 | 0.34 | 0.31 | 0.25 | 0.27 |
| **Depression** | 0.24 | 0.31 | 0.22 | 0.28 | 0.18 | 0.29 | 0.27 | 0.28 | 0.20 | 0.22 | 0.55 | **0.61** |
| **Distress** | 0.24 | 0.23 | 0.21 | 0.20 | 0.16 | 0.24 | 0.26 | 0.23 | 0.16 | 0.17 | 0.52 | 0.43 |
| **Vitality** | 0.45 | 0.55 | 0.58 | 0.55 | 0.25 | 0.42 | 0.46 | 0.49 | 0.52 | 0.55 | 0.42 | 0.54 |
| **Sexual activity** | 0.31 | 0.30 | 0.33 | 0.31 | 0.24 | 0.24 | 0.28 | 0.29 | 0.32 | 0.31 | 0.32 | 0.34 |
| **Overall 15D score** | 0.51 | **0.60** | **0.69** | **0.67** | 0.33 | 0.48 | 0.44 | 0.49 | 0.59 | 0.56 | 0.50 | 0.57 |

The moderately high correlations are bolded. The Spearman correlation coefficient values r <0.3 were considered poor, values 0.3 ≤ r < 0.6 fair, values 0.6 ≤ r < 0.8 moderately strong, and values r ≥ 0.8 very strong.

Abbreviations: PHQ-2, two-item Patient Health Questionnaire; RDS, Rose Dyspnea Scale; SAQ-AF, Seattle Angina Questionnaire Angina Frequency; SAQ-PL, Seattle Angina Questionnaire Physical Limitation; SAQ-QL, Seattle Angina Questionnaire Quality of Life; SAQ-7, Seattle Angina Questionnaire short-form; 15D, 15-dimensional instrument
